# Rapid and visual detection of *Toxoplasma gondii* oocyst in cat feces using loop-mediated isothermal amplification (LAMP) assay

**DOI:** 10.1038/s41598-023-44658-7

**Published:** 2023-10-12

**Authors:** Lingwei Sheng, Qiqi Xue, Sijia Xu, Fang Can, Ning Yao, Minghui Zou, Qiao Teng, Yuanyuan Li, Saeed El-Ashram, Yongsheng Ji, Jinhong Zhao

**Affiliations:** 1 Medical Laboratory Science, Wannan Medical College, Wuhu, 241002 Anhui China; 2 Department of Medical Parasitology, Wannan Medical College, Wuhu, 241002 Anhui China; 3 School of Public Health, Wannan Medical College, Wuhu, 241002 Anhui China; 4 College of Life Science and Engineering, Foshan University, Foshan, 528231 Guangdong China; 5 Faculty of Science, Kafrelsheikh University, Kafr El-Sheikh, 33516 Egypt; 6 Division of Life Sciences and Medicine, University of Science and Technology of China, Hefei, 230026 Anhui China; 7 Anhui Provincial Key Laboratory of Biological Macro-Molecules, Wuhu, 241002 Anhui China

**Keywords:** Parasitology, Infection

## Abstract

*Toxoplasma gondii* is an obligate parasitic protozoon that transmits to animals and humans via ingested food. Cats that act as *T. gondii’*s final hosts play a critical role in *T. gondii* transmission by shedding millions of oocysts. Timely diagnosis of infected cats is essential for preventing toxoplasmosis because oocysts are a putative *T. gondii* source in epidemiology. We developed a new visual LAMP assay targeting the B1 gene to analyze single oocysts in cat feces in this study. The amplification result could be visually estimated based on the color change. LAMP assay analytical sensitivity was 10^1^ copies/µL for the B1 gene plasmid, which was tenfold better than the PCR reaction. There were no cross-reactions with other parasites. The LAMP assay can detect a single *T. gondii* oocyst in 200 mg of cat feces. The LAMP assay detected a single oocyst in 200 mg cat feces at a higher rate than the PCR assay (83.3% vs. 50.0%).

## Introduction

*Toxoplasma gondii,* an obligate intracellular parasitic protozoon, is a global cause of food-borne zoonotic toxoplasmosis in humans and warm-blooded animals^1^. Those with healthy immune systems often show no symptoms or mild flu-like ones. Parasites may cause serious problems, including encephalitis and pneumonia, in persons who don't have functioning immune systems^2^. Pregnant women infected with this protozoan parasite may have a premature delivery, abortion, teratosis, or stillbirth, and neonates may develop ocular and neurological abnormalities^3^. *T. gondii* causes abortion, stillbirth, and deformities in sheep, pigs, and sows, resulting in major economic losses to the livestock sector^4–6^.

Cats, as the final hosts in the life cycle of *T. gondii*, play a critical role in transmitting *T. gondii* to animals and humans by excreting millions of oocysts and contaminating the environment. In humans and animals, *T. gondii* infection is generally induced by consuming tissue cysts from infected animal tissues and oocysts through contaminated water, soil, plants, or foods^4,7,8^. Oocysts discharged by stray and domestic cats are a source of *T. gondii* for people and animals in the epidemiology of this zoonotic parasite; consequently, rapid detection of infected cats is crucial to avoid the transmission of toxoplasmosis.

For decades, microscopy has been used to identify *T. gondii* oocysts in cat feces. Still, this method has low sensitivity, is time-consuming, is easy to miscalculate, and needs to be more accurate^9^. While serological assays are more sensitive than microscopy, the *T. gondii* antibody response often comes after the cat has finished excreting oocysts. It has some drawbacks, such as low sensitivity and the possibility of cross-reaction^10^. Various molecular biological methods, such as real-time PCR, nested PCR, and PCR, have gradually been introduced to diagnose *T. gondii* because of their higher sensitivity compared to bioassays^11,12^. However, there are drawbacks, including the high cost, the necessity of costly laboratory equipment, and the inability to be used in field conditions^13^. This highlights the need to develop efficient, accurate, and practical diagnostic procedures for early toxoplasmosis screening, prevention, and control.

Loop-mediated isothermal amplification (LAMP) is a molecular assay for amplifying specific DNA fragments^14^. LAMP has the advantages of being fast, specific, and sensitive, requiring only a thermostatic environment to perform amplification for 1 h and then detect the target gene. LAMP has shown high sensitivity and specificity for detecting *T. gondii*^13,15^. To identify *T. gondii* in blood samples, Mirahmadi et al. established a LAMP test targeting the B1 gene and having a higher sensitivity than nested PCR^16^. For the first time, Karakavuk et al. employed the LAMP assay to identify *T. gondii* in cat feces, assessing the amplified product through colorimetric and agarose electrophoresis. They revealed the LAMP test was just as sensitive as real-time PCR^11^. However, whether a single oocyst can be detected using the LAMP assay in cat feces has yet to be reported. Therefore, this research aimed to establish a B1-targeted rapid LAMP assay to detect *T. gondii* oocysts in cat feces. To verify the effectiveness of the LAMP assay established for upcoming clinical use, we assessed its sensitivity and specificity for B1 gene-positive plasmids and various numbers of oocysts in cat feces.

## Materials and methods

### Ethics approval

All animal care and experimental procedures were approved by the Laboratory Animal Committee of Wannan Medical College (#LLSC-2022-240) and were carried out according to rules and procedures of China National Institutes of Health for use of laboratory animals and guide for care and use of animals. The Study was carried out following ARRIVE guidelines.

### Collection of fecal samples and oocysts

The *T. gondii* Wh6 strain was isolated from the stray cat and routine preservation in our lab, the Department of Life Sciences and Medicine, University of Science and Technology of China. Female BALB/C mice infected with the *T. gondii* Wh6 strain had their brain tissue removed painlessly, gently crushed sterile to obtain a suspension in normal saline, and cysts were counted under a microscope. As described in our previous research, a healthy stray cat was given a suspension of brain tissue containing cysts^17^. After infection with *T. gondii*, cats were housed individually in stainless steel cages with restricted access, and feces were collected daily for one week. Oocysts were purified using sucrose flotation and a 2% H_2_SO_4_ suspension during oocyst shedding in cats^16^.

### Genomic template preparation and PCR amplification

The DNA of each parasite sample was extracted using the DNeasy Blood and Tissue Kit (Tiangen Biotechnology Beijing Co., Ltd., Beijing, China) per the manufacturer's directions. Fecal Kit extracted genomic DNA from feces (Tiangen Biotech Beijing Co., Ltd., Beijing, China). These DNA samples were stored at -20 ℃. Primers were used for B1 gene PCR amplification, F: 5′-GGGAGCAAGAGTTGGGACTA-3′ and R: 5′-CAGACAGCGAACAGAACAGA-3′^18^. The PCR reaction system included 12.5 µL Takara LA Taq (Takara Biotechnology Co., Ltd., Beijing, China), 1 µL F and R primers, 2 µL DNA template, and was replenished with ddH_2_O to 25 µL. The PCR amplification was performed under the following conditions: 10 min of initial denaturation at 95 ℃; 40 cycles of 30 s at 95 ℃, 30 s at 56 ℃ and 30 s at 72 ℃; and a final elongation step at 72 ℃ for 10 min. The PCR product was analyzed with 2% agarose gel electrophoresis.

### LAMP assay development

The B1 gene sequence of *T. gondii* (AF179871) was retrieved from the NCBI GenBank database to generate the B1 gene-specific LAMP primers^13^. Primer Explorer V4 software (http://primerexplorer.jp/lamp) was used to generate five different primers: an outer forward primer set (F3), an outer reverse primer set (B3), an inner forward primer set (FIP), an inner reverse primer set (BIP), and two loop primers (LF and LB). Each primer was commercially synthesized (Sangon Biotechnology Co., Ltd., Shanghai, China). The LAMP reaction was performed in a 25 µL reaction mixture by LAMP DNA Amplification Kit (Eiken Chemical Co., Ltd., Tochigi, Japan), including 12.5 µL RM, 40 pmol FIP, 40 pmol BIP, 20 pmol LF, 20 pmol LB, 5 pmol F3, 5 pmol B3, 1 µL FDR (Fluorescent visual reagent), 1 µL Bst DNA polymerase, 2 µL template genomic DNA and replenished with ddH_2_O. The LAMP reaction was conducted in a Loopamp real-time turbidimeter (LA-500; Eiken Chemical Co., Ltd., Tochigi, Japan) at 60–69 °C for 60 min, with 1 °C intervals. Finally, the reaction system was stopped by keeping the temperature at 80 °C for 5 min to inactivate the polymerase. The LAMP products were assessed utilizing a real-time turbidity detector to reveal the positive curve. Furthermore, fluorescent detection reagent technique for visual inspection was also performed. The LAMP product changed in color from colorless to green for a positive reaction, while the color did not become green and maintained colorless in the negative reaction. The color change is visible with the naked eye under natural light without additional instruments.

### LAMP assay specificity

To confirm the LAMP assay's B1 gene-based specificity for *T. gondii*, the DNA samples of *Digramma interrupta*, *Entamoeba coli*, *Vermivm terrestrium*, *Plasmodium vivax*, *Neospora caninum*, *Ascaris lumbricoides*, *Taenia solium, Schistosoma japonicum*, and *Trichinella spiralis* were chosen as control templates for the LAMP reaction. The parasites were received from the Parasitology Department at Wannan Medical College. Furthermore, the *T. gondii* B1 gene-positive plasmid was chosen as the positive group and ddH_2_O as the negative group.

### LAMP assay sensitivity

To assess *T. gondii* LAMP assay sensitivity using the B1 gene, the B1 gene-positive plasmid of *T. gondii* was serially diluted tenfold, from 10^3^ copies to 10^–2^ copies. Similar to our prior investigation, a positive plasmid carrying a 194 bp fragment of the B1 gene was developed^19^. The amplification findings were examined using the Loopamp real-time turbidimeter's turbidity graph and the color change of the reaction sample with the FDR.

### LAMP-based *T. gondii* oocyst detection

To confirm the LAMP assay established in this study, DNA was extracted from various numbers of *T. gondii* oocysts (1, 2, 3, 4, and 5 oocysts) utilizing a DNeasy Blood & Tissue Kit (Tiangen Biotech Beijing Co., Ltd., China), and the B1 gene was amplified using PCR and LAMP. The two approaches were used to assess these samples' positive detection rate and detection efficiency. Simulated clinical samples of *T. gondii*-infected cat feces were used to confirm the effectiveness of the LAMP assay. Different numbers of *T. gondii* oocysts, including 1, 2, 3, 4, and 5, were placed in 200 mg feces, and the DNA was extracted using the fecal kit (Tiangen Biotech Beijing Co., Ltd., Beijing, China). The standard of 200 mg feces was chosen because it was the maximum amount of fecal samples required by the fecal kit's instructions. These DNA samples were amplified and analyzed using PCR and LAMP assays to determine their validity for detecting oocysts in cat feces.

## Results and discussion

### Oocysts in cat feces

**Figure 1:**
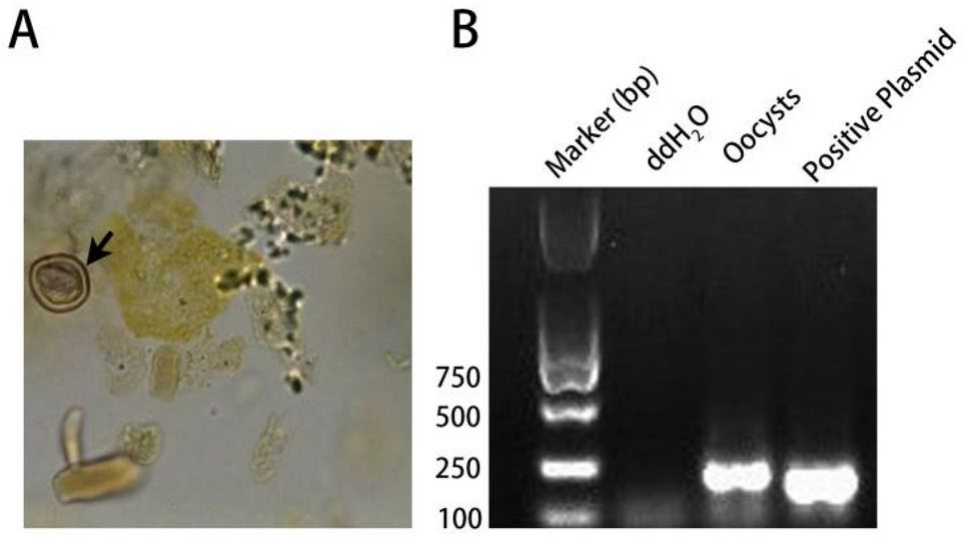
Identification of *T. gondii* oocysts. (**A**) Morphological characteristic of oocysts under 400× microscope. (**B**) Gel electrophoresis for amplification of oocysts by PCR. The arrow shows the oocyst.

*T. gondii* oocysts were observed under a microscope in cat feces after 4 days of infection with tissue cysts. The oocysts are subspherical to spherical and range in diameter from 8 to 12 µm. The oocyst wall was dense, allowing for little light transmission (Fig. 1A). Further PCR amplification and sequencing revealed that the *T. gondii* B1 target gene could be successfully amplified with the expected size (194 bp) from these oocyst samples (Fig. 1B), and BLAST analyses confirmed that the nucleotide sequence obtained was consistent with the known B1 gene sequence of *T. gondii* in GenBank.

### LAMP primers

**Figure 2:**
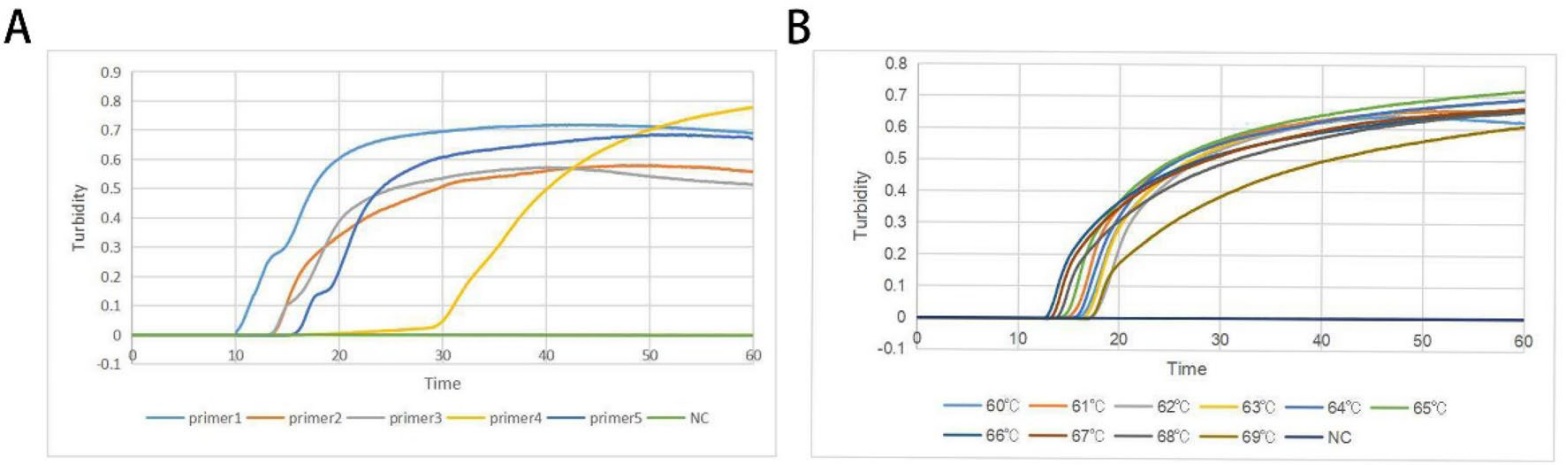
*T. gondii* B1 gene LAMP primer screening. (**A**) Turbidimetric curves of five primer pairs were monitored by a Loopamp real-time turbidimeter at 400 nm. (**B**) Turbidimetric curves of the temperature gradient (60–69 ℃) were monitored by a Loopamp real-time turbidimeter at 400 nm. The abscissa is the reaction time in minutes, and the ordinate is the real-time turbidity. NC means negative control of ddH_2_O.

The screening of primers was conducted with 10^3^ copies/μL of B1-positive plasmid as the DNA template at 60 °C using the Loopamp real-time turbidimeter. After 60 min, the LAMP reaction showed five distinct curves, showing that the five sets of primers had successfully amplified the target gene B1 in a specific manner. Because primer 1's curve appeared the fastest and had the best peak shape, it was chosen as the most effective primer for amplifying the target B1 gene and was used for *T. gondii* detection in subsequent experiments (Fig. 2A, Table 1).

**Table 1:** Primer sequences of the LAMP assay for targeting the *T. gondii* B1 gene.

Primer name	Primer sequence (5′-3′)
F3	GGGAGCAAGAGTTGGGACTA
B3	CAGACAGCGAACAGAACAGAA
FIP	CGCCTTTAGCACATCTGGTTCGAGATGCTCAAAGTCGACCGC
BIP	TATCGCAACGGAGTTCTTCCCAGGGCCTGATATTACGACGGAC
LF	CTTCTTCTGCGGGTGCATCT

### LAMP assay temperature

To optimize *T. gondii* B1 gene real-time LAMP assay reaction temperature, LAMP amplification was performed from 60 to 69 °C at intervals of 1 ℃ using the B1 positive plasmid (10^3^ copies/μL) as the template under the Loopamp real-time turbidimeter. We noticed that the best suitable reaction temperatures ranged from 60 to 69 °C, with robust amplification curves lasting between 10 and 20 min. The turbidity curve shows that the most amplified products were made when the amplification reaction was run at 65 °C (Fig. 2B). Thus, the optimal reaction temperature for amplifying the *T. gondii* B1 gene was 65 °C.

### LAMP assay specificity

**Figure 3:**
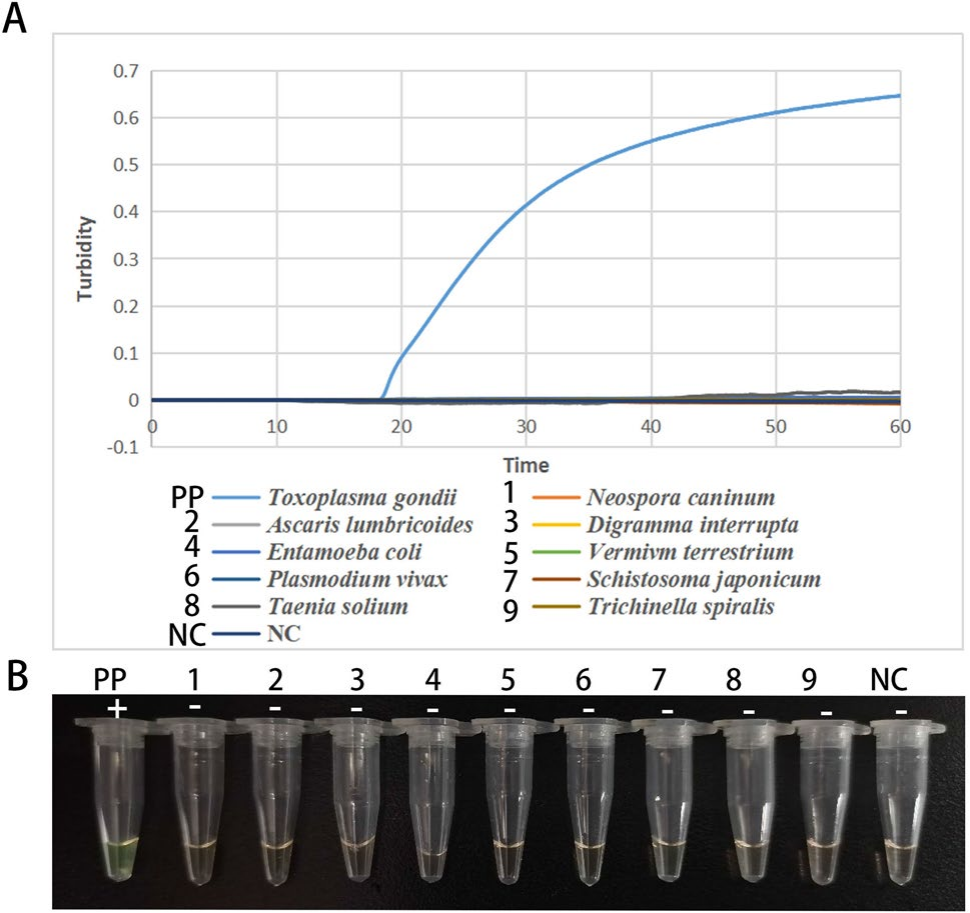
LAMP assay specificity for the *T. gondii* B1 gene*.* (**A**) real-time turbidity formation curves. The abscissa is the reaction time in min, and the ordinate is the real-time turbidity; (**B**) visible dye method for amplification by LAMP assay. PP means positive control of B1 positive plasmid, 1–9 means specific control samples, and NC means negative control of ddH_2_O. “ + ” means positive reaction, “−” means negative reaction.

The LAMP assay specificity was established employing other DNA samples, including *Neospora caninum*, *Digramma interrupta*, *Schistosoma japonicum*, *Ascaris lumbricoides*, *Vermivm terrestrium*, *Plasmodium vivax*, *Taenia solium, Entamoeba coli*, and *Trichinella spiralis.* The *T. gondii* B1 gene plasmid was used as a positive control, and ddH_2_O was used as a negative control. Except for the B1 gene-positive plasmid of *T. gondii*, no positive turbidity curve was observed in any of the above control samples using the turbidity method (Fig. 3A). The FDR method produced the same results as the real-time turbidity detection method. Only the positive plasmid group showed a green color (Fig. 3B). These results showed that the developed LAMP assay had a higher specificity for detecting *T. gondii* and that the LAMP assay produced was not a cross-reaction to the DNA of the nine samples mentioned above, that is the primer 1 designed in the current study is specific and effective for the detection of *T. gondii*.

### LAMP assay sensitivity

**Figure 4:**
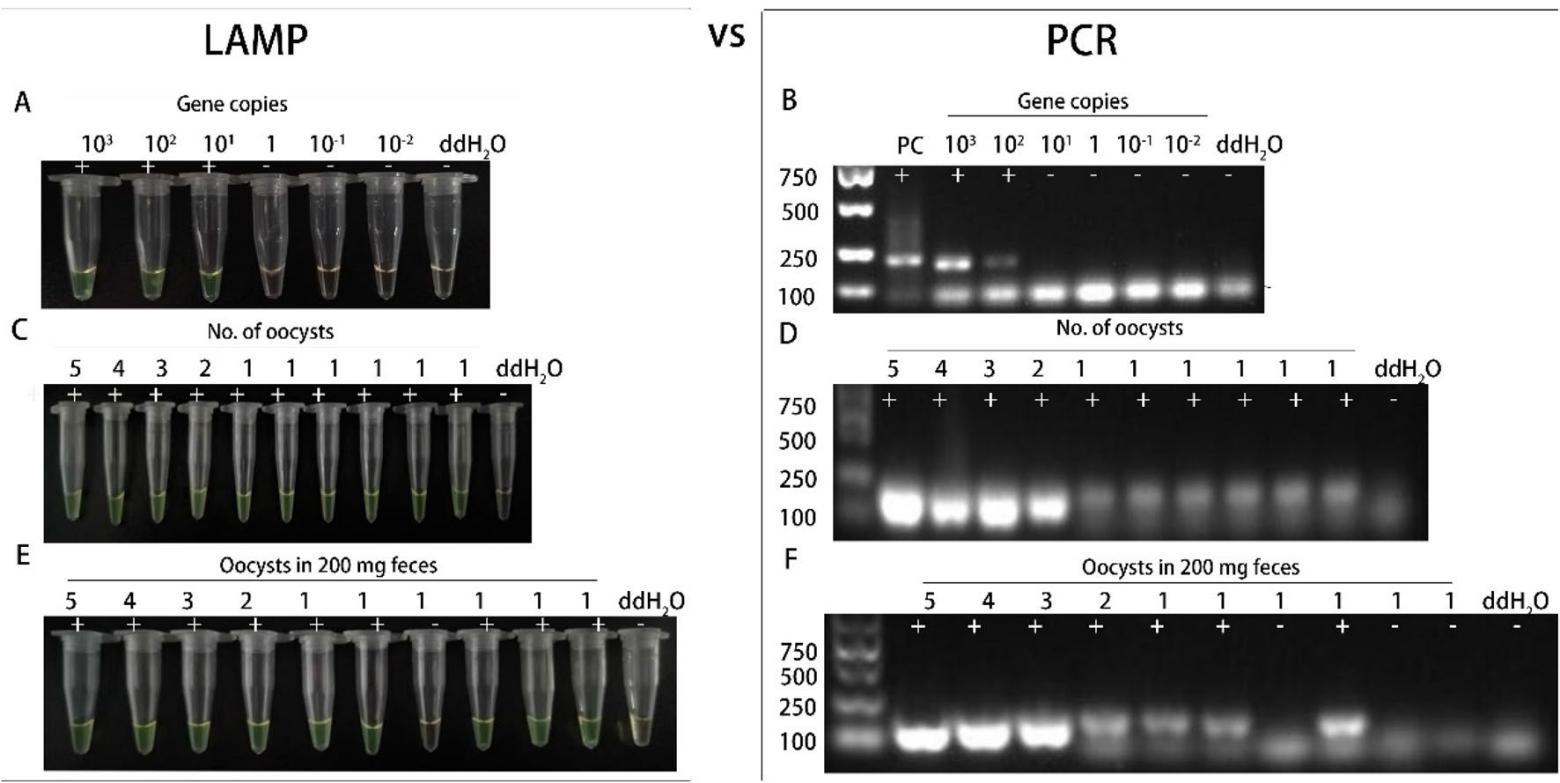
LAMP and PCR assay sensitivities for the *T. gondii* B1 gene (**A** and **B**) and oocysts of *T. gondii* (C to F). “ + ” means positive reaction, “−” means negative reaction.

Based on the turbidity curve, the established LAMP assay targeting the B1 gene has a minimum detectable concentration of 10^1^ copies/μL for positive plasmids (Fig. S1). The direct FDR visual method yielded the same outcome (Fig. 4A). We further validated the protocol using PCR and noticed that the minimum detection limit of the PCR method for the B1 gene was 10^2^ copies/μL. This allowed us to compare the LAMP amplification efficacy with traditional PCR (Fig. 4B). When traditional PCR was used as the reference assay, these results showed that the minimum detection limit of the LAMP assay was ten times higher than that of the PCR method. In a related study, LAMP tests targeting the *T. gondii* RE and B1 genes were 1000 and 100 times more sensitive than nested PCR^20^. Other research has also found that *T. gondii* LAMP assays are more sensitive than PCR methods^11,21,22^. Combined with the above specific analysis, the LAMP assays established in this study based on *T. gondii* B1 gene has good specificity and sensitivity for detecting *T. gondii.* As indicated in literature review, LAMP is considered to be one of the molecular diagnostic techniques, and it has proven to be highly sensitive and specific to detect *T. gondii*^21^.

### *T. gondii* oocyst LAMP efficiency

The LAMP assay sensitivity targeting the B1 gene for detecting *T. gondii* oocysts was assessed. When detecting different numbers of oocysts (5, 4, 3, 2, and 1) using the established LAMP assay, distinct turbidity curves appeared under the Loopamp real-time turbidimeter, even though a single oocyst was repeated six times to further validate the LAMP assay's efficiency (Fig.S1). The visual color method produced consistent results (Fig. 4C). These results illustrated the LAMP assay used in this investigation could detect a single oocyst, and the LAMP assay detection rate for a single oocyst is 100% (6/6), either the same detection rate of the traditional PCR (100%, 6/6) (Fig. 4D). To further validated the efficiency and the LAMP assay sensitivity newly established in this study, different numbers of oocysts (5, 4, 3, 2 and 1) were put into 200 mg cat feces (without infected *T. gondii*) to simulate the clinical samples infected with *T. gondii*. LAMP assay target B1 gene clinical sensitivity was also a single oocyst in 200 mg cat feces. When a single oocyst in 200 mg cat feces was tested six times to further validate the LAMP assay's efficiency, there were five positive results among six replicate groups, and the detection rate of a single oocyst in 200 mg feces by LAMP assay was 83.3% (5/6) (Fig. 4E). When traditional PCR was used as the reference method, the detection rate was 50% (3/6) (Fig. 4F).

Because of the large number of impurities and other substances in the cat fecal samples, the detection rate of LAMP and PCR decreased when detecting a single oocyst in cat fecal samples compared to a single oocyst, while the LAMP assay efficiency developed in this study was still better than PCR. This result also illustrate that LAMP assay was more sensitive than traditional PCR as the other research^21^.

*T. gondii* DNA was examined in blood and tissue samples from various humans and animals, as well as environmental water samples in some LAMP studies. Lalle et al. developed a LAMP method that detects *T. gondii* oocysts at concentrations as low as 25 oocysts per 50 g of ready-to-eat baby lettuce and five oocysts per mL of vegetable pellet suspension ^23^. In another study, 52 water samples were investigated utilizing LAMP, PCR, and the immunofluorescence test (IFT), and the *T. gondii* positive rate in these water samples was 48.1% (25/52), 13.5% (7/52), and 0% (0/52), respectively, demonstrating LAMP as a highly sensitive assay for detecting *T. gondii* in water samples^24^. In the current study, the LAMP assay can detect 1 oocyst per 200 mg of cat feces with the positive rate of 83.3% (5/6). These findings showed that LAMP assay protocol established in this study can be appropriate and optimal for detection of *T. gondii* infection in cat feces samples.

## Conclusion

In this study, we developed a novel visual LAMP assay targeting the B1 gene to evaluate a single oocyst in cat feces for the first time. Furthermore, we improved the visual LAMP assay's accuracy for evaluating amplification results with the naked eye versus the real-time turbidity detector. Which only needs an hour of operation in an isothermal environment, and the amplification effect is visible to the naked eye. Importantly, there is no need to remove the lid of the reaction tube to observe the results, and this can, to a certain degree, ovoid the aerosol pollution of the sample in LAMP amplification. The LAMP assay is more conducive to promotion and application in primary laboratories and on-site testing and has greater benefits for preventing the transmission of parasites from animals to humans.

### Supplementary Information

Supplementary Information 1.

Supplementary Figure 1.

## Data Availability

The datasets used and/or analysed during the current study available from the corresponding author on reasonable request. The B1 gene sequence of *T. gondii* (AF179871) was retrieved from the NCBI GenBank database.

## References

[1] Torgerson PR (2015). World health organization estimates of the global and regional disease burden of 11 foodborne parasitic diseases, 2010: A data synthesis. PLoS Med..

[2] Wang JL (2019). Advances in the development of anti-toxoplasma gondii vaccines: Challenges, opportunities, and perspectives. Trends Parasitol..

[3] Saadatnia G, Golkar M (2012). A review on human toxoplasmosis. Scand. J. Infect. Dis..

[4] Pan M, Lyu C, Zhao J, Shen B (2017). Sixty years (1957–2017) of research on toxoplasmosis in China—An overview. Front. Microbiol..

[5] Stelzer S (2019). Toxoplasma gondii infection and toxoplasmosis in farm animals: Risk factors and economic impact. Food Waterborne Parasitol..

[6] Katzer F (2011). Increased *Toxoplasma gondii* positivity relative to age in 125 Scottish sheep flocks; evidence of frequent acquired infection. Vet. Res..

[7] Kurth K, Jiang T, Muller L, Su C, Gerhold RW (2021). *Toxoplasma gondii* contamination at an animal agriculture facility: Environmental, agricultural animal, and wildlife contamination indicator evaluation. Int. J. Parasitol. Parasites Wildl..

[8] Shapiro K (2019). Environmental transmission of *Toxoplasma gondii*: Oocysts in water, soil and food. Food Waterborne Parasitol..

[9] Schares G, Vrhovec MG, Pantchev N, Herrmann DC, Conraths FJ (2008). Occurrence of *Toxoplasma gondii* and *Hammondia hammondi* oocysts in the faeces of cats from Germany and other European countries. Vet. Parasitol..

[10] Karakavuk M (2021). Investigation of the role of stray cats for transmission of toxoplasmosis to humans and animals living in Izmir, Turkey. J. Infect. Dev. Ctries..

[11] Karakavuk M (2022). Rapid detection of *Toxoplasma gondii* DNA in cat feces using colorimetric loop-mediated isothermal amplification (LAMP) assays targeting RE and B1 genes. Comput. Immunol. Microbiol. Infect. Dis..

[12] Salant H, Spira DT, Hamburger J (2010). A comparative analysis of coprologic diagnostic methods for detection of *Toxoplama gondii* in cats. Am. J. Trop. Med. Hyg..

[13] Xue Y (2021). A novel loop-mediated isothermal amplification-lateral-flow-dipstick (LAMP-LFD) device for rapid detection of *Toxoplasma gondii* in the blood of stray cats and dogs. Parasite.

[14] Notomi T (2000). Loop-mediated isothermal amplification of DNA. Nucl. Acids Res..

[15] Sun XM (2017). Improvement and evaluation of loop-mediated isothermal amplification for rapid detection of *Toxoplasma gondii* infection in human blood samples. PLoS ONE.

[16] Sokol SL, Wong ZS, Boyle JP, Dubey JP (2020). Generation of *Toxoplasma gondii* and *Hammondia hammondi* oocysts and purification of their sporozoites for downstream manipulation. Methods Mol. Biol..

[17] Jin Y (2019). The Neurotropic parasite *Toxoplasma gondii* induces astrocyte polarization through NFkappaB pathway. Front. Med. (Lausanne).

[18] NasiruWana M (2020). Molecular detection and genetic diversity of *Toxoplasma gondii* oocysts in cat Faeces from Klang Valley, Malaysia, using B1 and REP Genes in 2018. Pathogens.

[19] Zhao J (2022). A novel rapid visual detection assay for *Toxoplasma gondii* combining recombinase-aided amplification and lateral flow dipstick coupled with CRISPR-Cas13a fluorescence (RAA-Cas13a-LFD). Parasite.

[20] Fallahi S, SeyyedTabaei SJ, Pournia Y, Zebardast N, Kazemi B (2014). Comparison of loop-mediated isothermal amplification (LAMP) and nested-PCR assay targeting the RE and B1 gene for detection of *Toxoplasma gondii* in blood samples of children with Leukaemia. Diagn. Microbiol. Infect. Dis..

[21] Hegazy MK, Awad SI, Saleh NE, Hegazy MM (2020). Loop mediated isothermal amplification (LAMP) of *Toxoplasma* DNA from dried blood spots. Exp. Parasitol..

[22] Wu Y (2022). Rapid and visual detection of *Toxoplasma gondii* in blood samples from pet cats and dogs by loop-mediated isothermal amplification. Vector Borne Zoonotic Dis..

[23] Lalle M, Possenti A, Dubey JP, Pozio E (2018). Loop-mediated isothermal amplification-lateral-flow dipstick (LAMP-LFD) to detect *Toxoplasma gondii* oocyst in ready-to-eat salad. Food Microbiol..

[24] Sotiriadou I, Karanis P (2008). Evaluation of loop-mediated isothermal amplification for detection of *Toxoplasma gondii* in water samples and comparative findings by polymerase chain reaction and immunofluorescence test (IFT). Diagn. Microbiol. Infect. Dis..

