# Supplementary material for: Rapid and visual detection of Toxoplasma gondii oocyst in cat feces using loop-mediated isothermal amplification (LAMP) assay
Source: Sci Rep. 2023 Oct 12;13:17269. doi: 10.1038/s41598-023-44658-7 (PMC10570283; doi:10.1038/s41598-023-44658-7)

Monad
莫纳生物

MonTrack™ D2000 DNA Ladder

REF：ME40601

结果展示

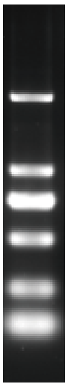

bp	ng/5 μl
2000	50
1000	50
750	150
500	50
250	50
100	50

2.0% 琼脂糖凝胶电泳结果

上样量:5 μl

电泳条件:

- 电压 8 V/cm
- 1× TAE
- 电泳 30 min

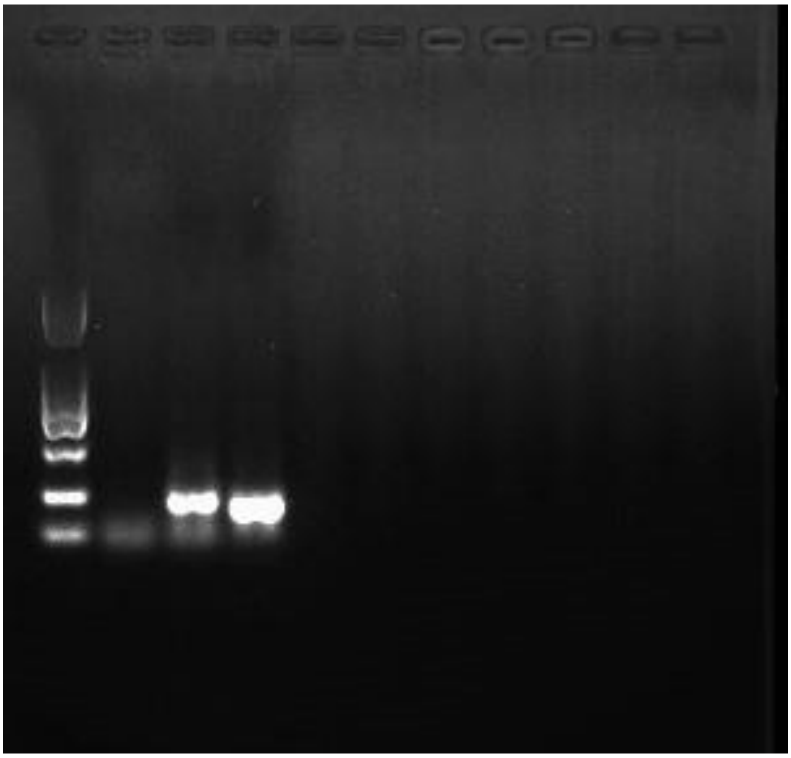

Raw image for Fig. 1B

After brightness/contrast adjustment

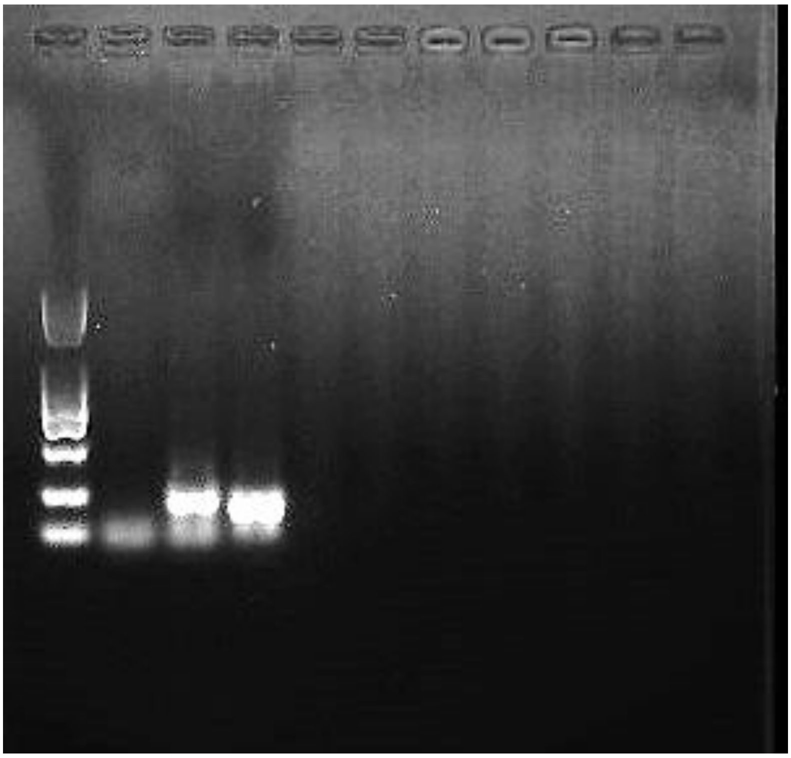

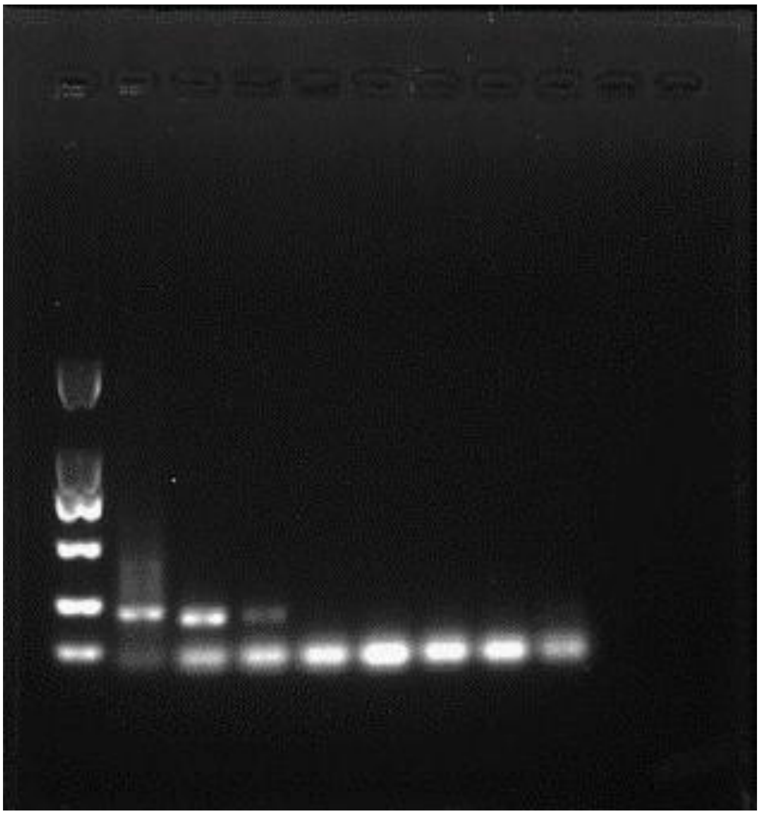

Raw image for Fig. 4B

After brightness/contrast adjustment

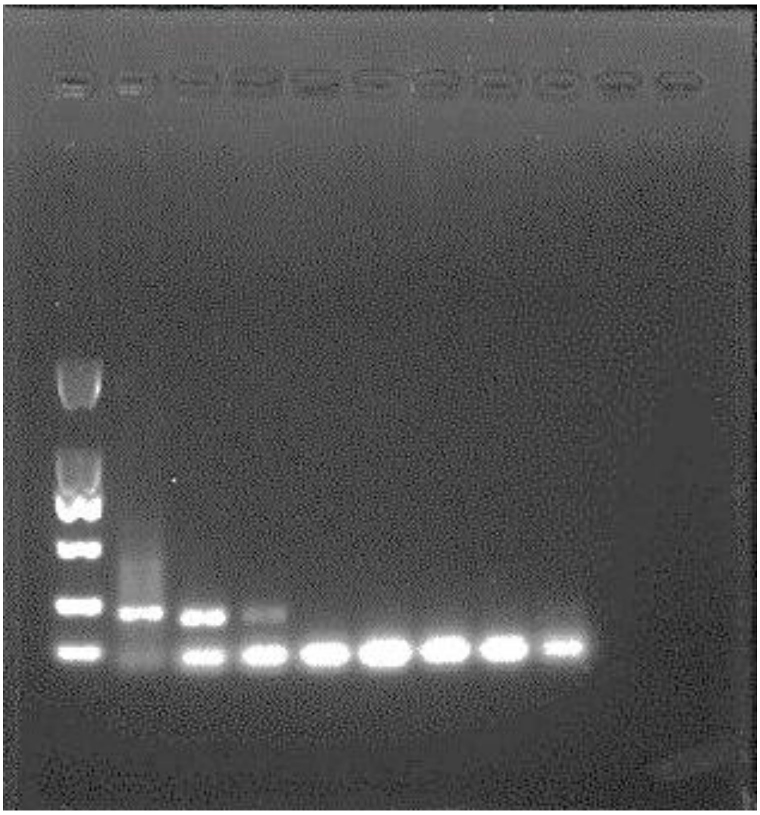

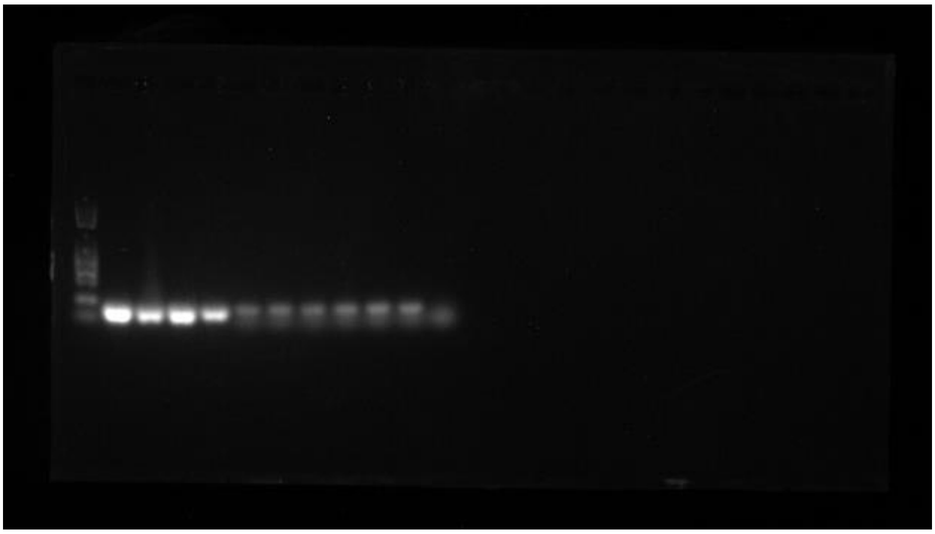

Raw image for Fig. 4D

After brightness/contrast adjustment

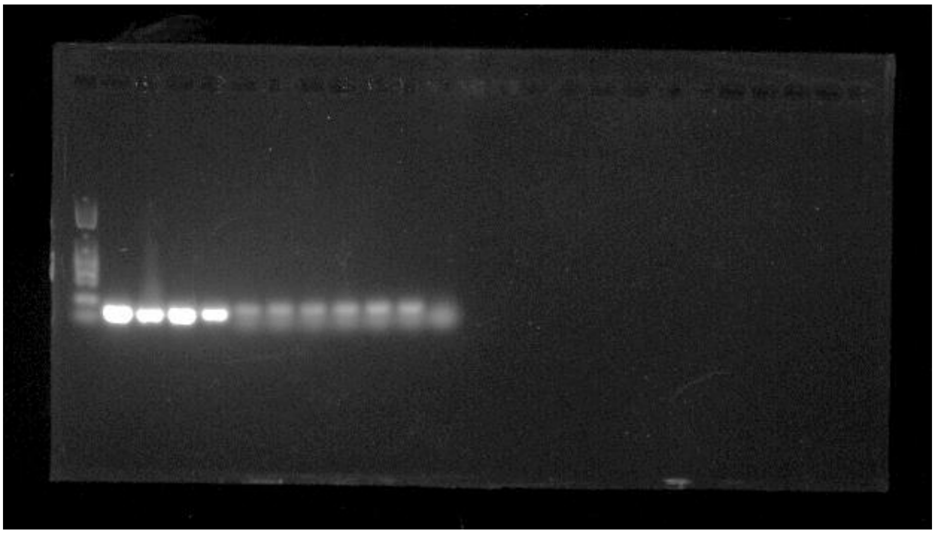

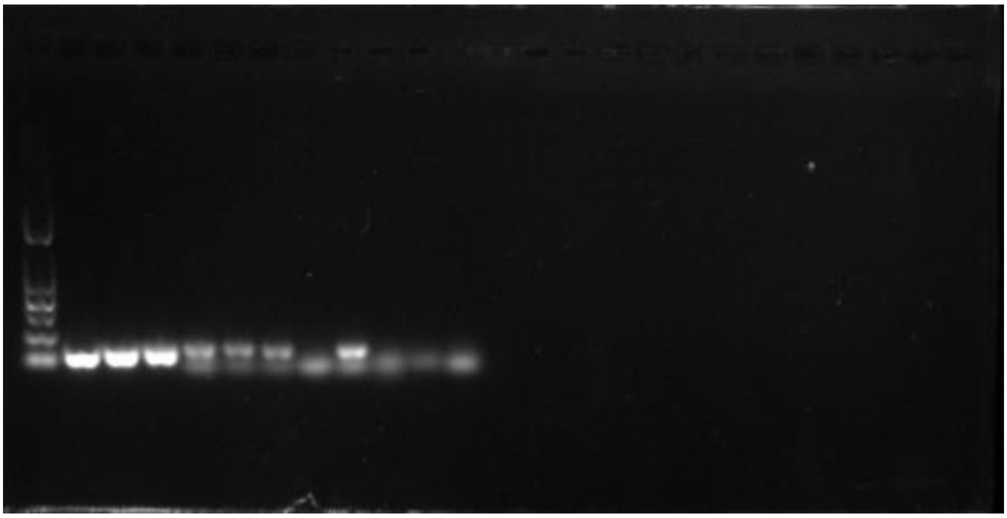

Raw image for Fig. 4F

After brightness/contrast adjustment

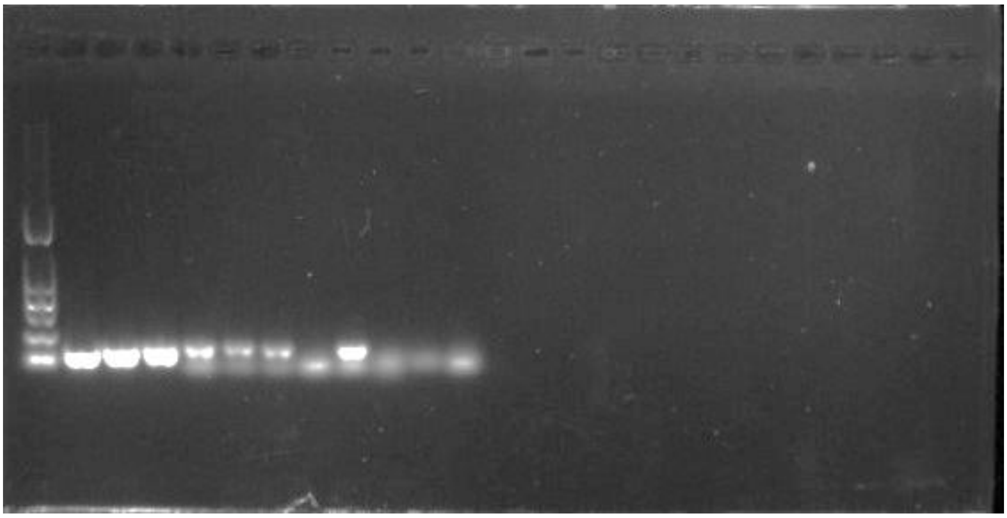

Supplement: Supplementary file 1 — Supplementary Information 1. [file 41598_2023_44658_MOESM1_ESM.pdf]
