# Supplementary figures and images for: Rapid and visual detection of Toxoplasma gondii oocyst in cat feces using loop-mediated isothermal amplification (LAMP) assay
Source: Sci Rep. 2023 Oct 12;13:17269. doi: 10.1038/s41598-023-44658-7 (PMC10570283; doi:10.1038/s41598-023-44658-7)

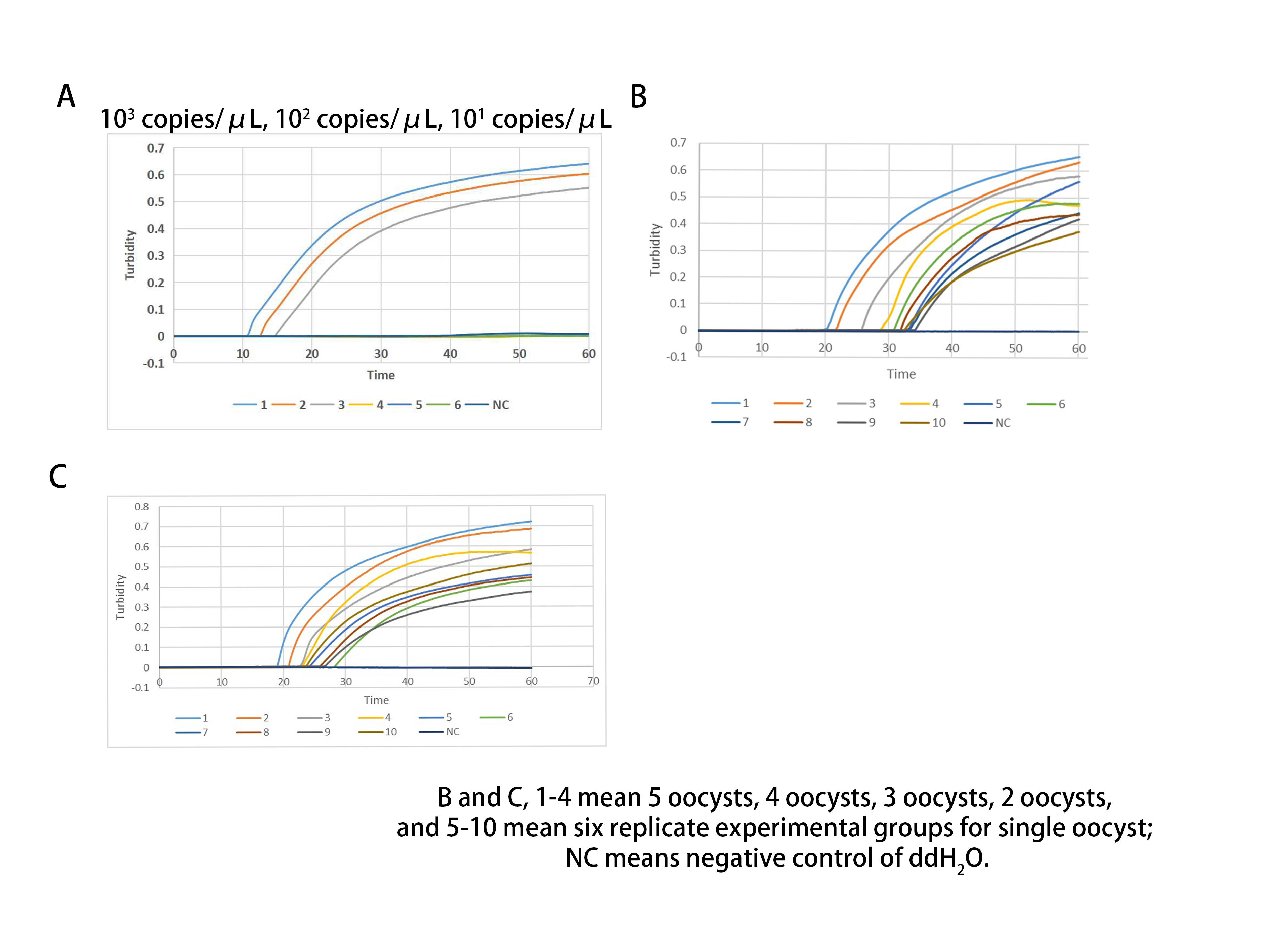

Supplement: Supplementary file 2 — Supplementary Figure 1. [file 41598_2023_44658_MOESM2_ESM.jpg]
